# Detection of viral antigen, IgM and IgG antibodies in cerebrospinal fluid of Chikungunya patients with neurological complications

**DOI:** 10.1186/1743-8454-7-12

**Published:** 2010-08-13

**Authors:** Rajpal S Kashyap, Shweta H Morey, Nitin H Chandak, Hemant J Purohit, Girdhar M Taori, Hatim F Daginawala

**Affiliations:** 1 Biochemistry Research Laboratory, Central India Institute of Medical Sciences, 88/2, Bajaj Nagar, Nagpur 440010, India; 2 Environmental Genomic Unit, National Environmental Engineering Research Institute, Nehru Marg, Nagpur 440020, India

## Abstract

**Background:**

During Chikungunya virus (CHIKV) epidemic in Nagpur, India, we identified some suspected Chikungunya patients with neurological complications. Early and cost-effective diagnosis of these patients remains problematic despite many new advanced diagnostic methods. A reliable diagnostic test, which could be performed in any standard pathology laboratory, would help to obtain definitive early diagnosis of CHIKV patients with neurological complications. In our laboratory, in-house ELISA protocol for viral antigen, immunoglobulin M (IgM) and IgG detection has been developed and assessed for the diagnosis of CHIKV patients with neurological complications.

**Method:**

Cerebrospinal fluid samples of forty-six patients who developed neurological symptoms within two months of CHIKV infections along with control subjects were included in the study and were analyzed for the presence of antigens and of IgM and IgG using an ELISA protocol.

**Results:**

The ELISA method for antigen detection yielded 80% sensitivity and 87% specificity for the diagnosis of CHIKV patients with neurological complications. The sensitivity for detection of IgM 48% or IgG 63% was significantly lower than the antigen assay (80%).

**Conclusion:**

The detection of viral antigen in CSF of CHIKV patients with neurological complications by ELISA method gave a more reliable diagnosis than antibodies detection that can be used to develop an immunodiagnostic assay with increased sensitivity and specificity.

## Background

Chikungunya virus (CHIKV) is an insect-borne virus, of the genus, *Alphavirus*, that is transmitted to humans by virus-carrying *Aedes *mosquitoes [1]. There have been recent outbreaks of CHIKV associated with severe morbidity. CHIKV causes an illness with symptoms similar to dengue fever and manifests itself with an acute febrile phase of the illness which lasts only 2-5 days, followed by a prolonged arthralgic disease that affects the joints of the extremities [2]. The pain associated with CHIKV infection of the joints persists for weeks or months and in some cases even for years.

In 2006, an outbreak was recorded in different areas of Maharashtra state. Over 2000 cases of Chikungunya fever have been reported from Malegaon town in Nasik district, Maharashtra state, India between February-March 2006 [3]. In May 2006, a big outbreak in Nagpur district of Maharashtra was also reported. In Nagpur city alone, as per communication of District Health Officer, in Government dispensaries 50 to 100 cases of Chikungunya were seen everyday. Patients treated by private medical practitioners were not included in these numbers [4]. Some deaths have been reported, but these have been attributed mainly to inappropriate management. The major causes of morbidity included severe dehydration, electrolyte imbalance, and loss of glycemic control.

During the epidemic, in our institute we have identified several suspected CHIKV patients associated with neurological complications [5]. Our Institute is the primary referral centre for neurological illnesses and serves as tertiary referral centre for patients not only from Vidarbha (Part of Maharashtra, India), but also from adjoining states like Madhya Pradesh, Chhattisgarh and Andhra Pradesh. To our knowledge, only a few CHIKV-associated neurological manifestations have been reported previously in the Indian subcontinent. However recently, some cases of CHIKV meningoencephalitis and myelopathy were reported from the Reunion Islands [6,7].

Monitoring the clinical features of CHIKV infection is an important component of assessing the disease process in humans so as to determine which organ system including the nervous system is affected. Laboratory tests are necessary to confirm the diagnosis of CHIKV. The immunoglobulin M (IgM) capture ELISA method, which provides evidence of CHIKV infection, is widely used to lend support to clinical findings in the assessment of patients with suspected CHIKV [8]. However, the sensitivity of IgM capture ELISA is low in the majority of patients in the acute stage (days 1-5); therefore, a negative IgM capture ELISA does not rule out the diagnosis. The specificity of IgM capture ELISA is also limited because of cross-reactivity with dengue or other infections. IgG cannot be detected in CHIKV patients in the acute stage.

A rapid antigen detection test using ELISA for CHIKV infection may be a more accurate diagnostic method for CHIKV patients with associated with neurological complications. In our laboratory, we have developed an ELISA based viral antigen detection assay for the diagnosis of CHIKV infection. The test was evaluated in serum samples from CHIKV patients with a sensitivity and specificity of 85% and 89% **(9)**. In the present study, cerebrospinal fluid (CSF) specimens from CHIKV patients associated with neurological complications been examined by a sensitive and specific indirect ELISA protocol developed in our laboratory. The purpose of the study was to evaluate the sensitivity and specificity of rapid antigen ELISA compared to IgG and IgM tests and also to determine their efficacy in absence of molecular techniques.

## Materials and methods

### Study subjects

Three hundred patients of clinically-suspected CHIKV infections were enrolled for the study from July-December 2006 at the Central India Institute of Medical Sciences (CIIMS), Nagpur, India. All patients had typical clinical features of CHIKV infection fever, headache, body ache, myalgia, and joint pains with or without swelling and without hemorrhagic rash. Forty-six patients who developed neurological symptoms were hospitalized. Patients showing any neurological symptoms (encephalitis, myelopathy, neuropathy, myeloneuropathy or myopathy) along with the typical Chikungunya infection symptoms i.e. joint pain with or without swelling, rashes and fever were considered for the study. Any patients presenting pain clearly related to other etiologies such as rheumatologic, muscular, migraines, diabetes, psychiatric illness etc were excluded since they could confound the analysis. Patients with entrapment neuropathy were also excluded from the study. All had detailed neurological evaluation and blood investigations. Depending on the neurological syndrome, patients were further investigated using CSF analysis, electrodiagnostic tests, electroencephalography (EEG), evoked potential studies, and neuroimaging, computerized tomograpy (CT) and/or magnetic resonance imaging (MRI). Molecular tests reverse transcriptase polymerase chain reaction, [RT-PCR], real-time PCR and virus isolation were done in all the patients included in the present study, first in serum samples and then in CSF.

Neurological infection of CHIKV was confirmed if reverse transcription polymerase chain reaction (RT-PCR) and/or real time PCR or virus isolation in CSF samples were positive and were included in the confirmed Neuro-CHIKV group. When all these tests were negative, the patients were diagnosed by above mentioned clinical symptoms and formed the suspected neuro-CHIKV group. Patients were given supportive care as per the clinical syndrome and the course of the illness. Some received a course of methyl prednisolone 1g daily for 3-5 days. Fifteen patients, who had no clinical features of Chikungunya and had no evidence of CNS or extra-CNS infections, and were negative for PCR or virus isolation, were grouped in the Non-CHIKV and analyzed as a control group. The median age of the study subjects was 45 years (range, 5-85 years). The Institutional Ethics Committee of the CIIMS, Nagpur, India approved the study.

### CSF sampling and handling

CSF was collected by standard lumbar puncture. Approximately 5-10 ml CSF was obtained, part of it was used for routine biochemical and pathological analysis including gram staining, India ink staining and AFB staining and culturing and the remaining CSF was used for the ELISA study. CSF samples were collected from all study groups for which patients' written consent was obtained. All the samples were stored at 4°C until further analysis.

### CHIKV antigen and antibodies

The CHIKV antigen used in the ELISA test was prepared using an Indian strain of Chikungunya virus CHIKV ISW HYD06 (Gene Bank accession number 876190). Pooled sera from CHIKV patients was collected, and IgG (antibodies) was purified by protein G affinity-column chromatography (IgG purification kit, Bangalore Genei, India) according to the manufacturer's instructions and used at a dilution of 1:10,000.

### ELISA (CHIKV antigen detection)

CSF samples (100 μl, diluted 1:5) from CHIKV patients who have neurological complications were added to the microtiter wells, incubated for 60 min and then blocked with 0.5% BSA in PBS-T (phosphate buffered saline - tween-20) for 60 min. After the samples were washed with PBS-T, anti-CHIKV IgG was added (1:10,000) and the plates were incubated at 37°C for 60 min. After incubation, wells were washed and goat-anti-human IgG-HRP (Horseradish peroxidase) secondary antibody (1:10,000 dilution) was added. Then samples were incubated for 60 min at 37°C. After another wash with PBS, 100 μl of TMB/H_2_O_2 _(tetramethylbenzidine - hydrogen peroxide) substrate solution was added to the wells, which were incubated at room temperature for approximately 10 min. The reaction was then terminated with 100 μl of 2.5 N H_2_SO_4_. The absorbance of each well was read at 450 nm.

### ELISA (CHIKV antibodies detection)

The immunoglobulin (IgM) and IgG antibodies to CHIKV were detected in CSF by ELISA. The ELISA protocol detected IgG and IgM antibodies directed against CHIKV antigens. The viral antigens (1:2 dilution) in PBS-T were coated onto the microtitre wells. After 90 min of incubation at room temperature, the wells were washed with PBS-T, and after blocking with 0.5% BSA in PBS-T for 60 min, CSF samples (1:5 dilution) were added to the wells and incubated for 60 min. After the incubation, 100 μl of goat anti-human IgG or IgM conjugated with horseradish peroxidase was added. After another washing with PBS, 100 μl of TMB/H_2_O_2 _substrate solution was added to the wells, which were incubated at room temperature for approximately 10 min. The reaction was terminated with 100 μl of 2.5 N H_2_SO_4_. The absorbance of each well was read at 450 nm.

### Statistical analysis

The results were expressed as the mean ± standard deviation (SD) together with the range. Comparisons of the proportions of positive results were made between the different tests and subgroups by using the Kruskal-Wallis test (nonparametric analysis of variance) with Dunnett's post test. A *P *value of less than 0.05 was considered significant. The sensitivity (true-positive rate) for the test was calculated as [the number of samples in the CHIKV-infected group with an absorbance of greater than or equal to the (mean + SD) of the absorbance for the healthy group divided by the total number of samples for the CHIKV-infected group] X100. The specificity (true-negative rate) for the test was calculated as [the number of samples in the CHIKV-infected group with an absorbance less than the (mean + SD) of the absorbance for the healthy group divided by the total number of samples for the healthy group] × 100.

## Results

Three hundred patients of clinically suspected CHIKV infections were seen during the period of June 2006 to December 2006 and followed up for any neurological complications until April 2007. Forty six out of 300 patients enrolled for the study developed neurological complications. Among them, 39 were males and 7 were females. With one exception, all patients developing neurological complications were over 20 years of age. The majority of the patients developed neurological complications within 20 days of onset of CHIKV infection. Thirty-three out of 46 patients were given corticosteroid treatment and remaining kept on supportive treatment. All the patients improved irrespective of type of treatment except 2 patients; one from the encephalitic group and another from myeloneuropathy group expired.

### CHIKV antigen assay

**Figure 1:**
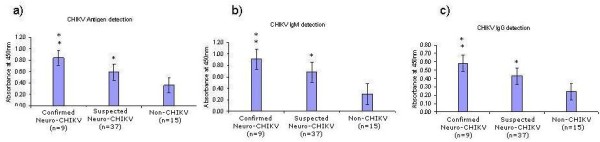
**Mean absorbance values of ELISA assays for a) CHIKV viral antigen, b) CHIKV IgM, c) CHIKV IgG in CSF from patients with confirmed (n = 9) and suspected CHIKV (n = 37) with neurological complications and patients from non-CHIKV group (n = 15)**. Data are means +/- SD. * *P *< 0.05, ** *P *< 0.01 relative to control non-CHIKV subjects.

The CSF positivities for CHIKV antigen in cases of confirmed neuro-CHIKV and suspected patients were 100% (9/9) and 76% (28/37) respectively, while the positivity for patients in the Non-CHIKV group was 13% (02/15) (Table 1). The mean absorbance value of CHIKV antigen in CSF from the CHIKV and non CHIKV groups as determined by the ELISA method is shown in Figure 1A. The mean absorbance value of CHIKV antigen in the confirmed neuro-CHIKV patients was 0.84 ± 0.19 and for suspected Chikungunya patients was 0.64 ± 0.19 which were significantly higher than the non CHIKV group (0.36 ± 0.08; *P *< 0.001). There was a significant difference in the mean CHIKV antigen activity between the confirmed neuro-CHIKV patients (0.84 ± 0.23) and the suspected-CHIKV patients (0.59 ± 0.14; *P *< 0.05).

**Table 1:** Demonstration of CHIKV antigen in CSF of Neuro-CHIKV and Non-CHIKV group by ELISA method.

Study Subjects	Positivity for CHIKV Antigen (%)	Negativity for CHIKV Antigen (%)
**Neuro-CHIKV patients (n = 46)**	37 (80%)	9 (20%)
**Confirmed (n = 9)**	9 (100%)	0
Encephalitis (n = 4)	4 (100%)	0
Myelopathy (n = 2)	2 (100%)	0
Myeloneuropathy (n = 1)	1 (100%)	0
Peripheral Neuropathy (n = 1)	1 (100%)	0
Myopathy (n = 1)	1 (100%)	0
**Suspected (n = 37)**	28 (76%)	9 (24%)
Encephlitis (n = 20)	16 (80%)	4 (20%)
Myelopathy (n = 5)	5 (100%)	0
Myeloneuropathy (n = 6)	4 (67%)	2 (33%)
Peripheral Neuropathy (n = 6)	3 (50%)	3 (50%)

**Non-CHIKV patients (n = 15)**	2 (13%)	13 (87%)

### IgM assay

Table 2 shows the positivity for IgM assay in cases of confirmed neuro-CHIKV patients, suspected CHIKV patients and non CHIKV group. The CSF positivity for CHIKV IgM in confirmed CHIKV patients was 89% (8/9) and 38% (14/37) for the suspected CHIKV patients while the positivity for patients in the non CHIKV group was 27% (04/15). The mean IgM level of 0.85 ± 0.16 in the confirmed neuro-CHIKV group and 0.68 ± 0.16 in the suspected neuro-CHIKV group were significantly higher than the non CHIKV group 0.30 ± 0.07, *P *< 0.01 and 0.05 respectively (Figure 1B).

**Table 2:** Demonstration of CHIKV IgM in CSF of Neuro-CHIKV and Non-CHIKV group by ELISA method.

Study Subjects	Positivity for IgM (%)	Negativity for IgM (%)
**Neuro-CHIKV patients (n = 46)**	22 (48%)	24 (52%)
**Confirmed (n = 9)**	8 (89%)	1 (11%)
Encephalitis (n = 4)	3 (75%)	1 (25%)
Myelopathy (n = 2)	2 (100%)	0
Myeloneuropathy (n = 1)	1 (100%)	0
Peripheral Neuropathy (n = 1)	1 (100%)	0
Myopathy (n = 1)	1 (100%)	0
**Suspected (n = 37)**	14 (38%)	23 (62%)
Encephlitis (n = 20)	5(25%)	15 (75%)
Myelopathy (n = 5)	3 (60%)	2 (40%)
Myeloneuropathy (n = 6)	4 (67%)	2 (33%)
Peripheral Neuropathy (n = 6)	2 (33%)	4 (67%)

**Non-CHIKV patients (n = 15)**	4 (27%)	11 (73%)

### IgG assay

IgG detection in confirmed and suspected neuro-CHIKV patients were 56% (5/9) and 65% (24/37) respectively as given in Table 3. The IgG positivity for patients in the non CHIKV group was quite high i.e. 53% (8/15). Figure 1C shows the mean absorbance value of IgG in confirmed neuro-CHIKV group was 0.56 ± 0.08 and in suspected neuro-CHIKV was 0.43 ± 0.08, both were significantly higher than the non-CHIKV group 0.24 ± 0.08, *P *< 0.01 and 0.05, respectively.

**Table 3:** Demonstration of CHIKV IgG in CSF of Neuro-CHIKV and Non-CHIKV group by ELISA method.

Study Subjects	Positivity for IgG (%)	Negativity for IgG (%)
**Neuro-CHIKV patients (n = 46)**	29 (63%)	17 (37%)
**Confirmed (n = 9)**	5 (56%)	4 (44%)
Encephalitis (n = 4)	1 (25%)	3 (75%)
Myelopathy (n = 2)	1 (50%)	1 (50%)
Myeloneuropathy (n = 1)	1 (100%)	0
Peripheral Neuropathy (n = 1)	1 (100%)	0
Myopathy (n = 1)	1 (100%)	0
**Suspected (n = 37)**	24 (65%)	13 (35%)
Encephalitis (n = 20)	15 (75%)	5 (25%)
Myelopathy (n = 5)	4 (80%)	1 (20%)
Myeloneuropathy (n = 6)	1 (17%)	5 (83%)
Peripheral Neuropathy (n = 6)	4 (67%)	2 (33%)

**Non-CHIKV patients (n = 15)**	8 (53%)	7 (47%)

Overall, our study suggests that the antigen detection in both confirmed as well as suspected neuro-CHIKV groups was positive in the majority of patients (80%), while this was not the case for detection of IgM (48%) or IgG (63%).

## Discussion

The first report on CHIKV infection with neurological complication was reported in the 1960 s. There were some reports which suggest that this infection was found to be associated with meningoencephalitis, myelitis, and choroiditis [9,10]. In the recent epidemic various neurological complications have been reported which include meningo-encephalitis, meningo-encephalo-myeloradiculitis, myeloradiculitis, myelitis, myeloneuropathy, Guillain-Barre' syndrome, external opthalmoplegia, facial palsy, sensorineural deafness, and optic neuritis. As reported by others and from our laboratory encephalitis appears to represent the most common clinical manifestation and occurs either simultaneously or within few days of onset of systemic symptoms, during the period of viremia [5,9].

Molecular tests have also been developed but these methods require sophisticated technology and well-trained personnel. In addition, viral RNA is not usually detected in the serum after defervescence [11]. In addition to that, various immunoassays for detecting antibodies (IgG and IgM) in serum samples have been used [12-14]. However, despite extensive work on the CHIKV, diagnosis the sensitivity and specificity of these methods are limited. Our goal was to determine whether antigen detection instead of antibody detection improves the diagnostic assessment of patients with CHIKV infection in routine medical practice. ELISA-based CHIKV antigen detection which was developed in our laboratory was evaluated in the CSF collected from suspected cases of CHIKV infection with neurological complications along with a control group.

The data demonstrate that the positivities for CHIKV antigen in CSF from cases of confirmed and clinically-diagnosed CHIKV patients with neurological complications were better than the IgM and IgG detection method. To the best of our knowledge, this is the first ELISA-based antigen detection method reported and the first study that reports a comparison between antigen and antibody detection in the same CSF samples. Hundekar *et al *have developed a monoclonal antibody-based antigen capture ELISA to detect Chikungunya virus antigen in mosquitoes, but not in body fluids [15]. There are many studies on IgG and IgM analysis performed in serum samples of Chikungunya patients. Suryawanshi *et al *have done IgM analysis in 166 serum samples selected from suspected and confirmed cases of Chikungunya patients. Out of that 87 (52.4%) were positive for the developed test [16] .

The antigen detection assay which we have developed in our laboratory measures the viral protein in the CSF, which is detectable earlier than IgG and IgM during acute infection. This early detection is due to the initial burst of virus replication and is associated with high levels of viremia, and during which time the individual is highly infectious.

## Conclusions

The diagnostic sensitivity of CHIKV antigen detection using an ELISA-based system is higher than that of conventional IgM and IgG tests and helpful in the detection of antigen throughout the infection, even in the earlier stages of infection. The ELISA method used in this study is sensitive, specific, rapid, and cost-effective, and it can be adopted by any laboratory with an ELISA reader and an incubator. It may thus be useful in laboratories with limited resources, especially in underdeveloped and developing countries.

## Competing interests

The authors declare that they have no competing interests.

## Authors' contributions

RSK and SHM carried out the study design, data collection, statistical analysis, data interpretation, literature search, and manuscript preparation; NHC and HJP participated in the study design, data collection and preparation of the manuscript; GMT provided assistance in preparation of the manuscript, data collection and interpretation, study design, and funds collection; and HFD supervised the study design, statistical analysis, data interpretation, manuscript preparation, and literature search. All authors have read and approved the final version of the manuscript.
